# Supplementary material for: A Structure-Based B-cell Epitope Prediction Model Through Combing Local and Global Features
Source: Front Immunol. 2022 Jul 1;13:890943. doi: 10.3389/fimmu.2022.890943 (PMC9283778; doi:10.3389/fimmu.2022.890943)
Supplement: Supplementary file 1 [file Table_1.pdf]

PDB ID	Ag Size	DiscoTope-2.0			EpiPred			Our model		
		Precision	Recall	MCC	Precision	Recall	MCC	Precision	Recall	MCC
4hj0	92	0	0	0	0.32	**0.9**	**0.27**	**0.61**	0.621	0.231
1tzh	94	0.73	**0.87**	**0.72**	0.01	0.06	0.04	**0.692**	0.683	0.376
4am0	96	0.33	0.2	**0.19**	0.13	**0.7**	0.09	**0.547**	0.569	0.114
2ih3	97	0	0	0	0.16	0.64	0.08	**0.755**	**0.765**	**0.519**
4i77	97	0	0	0	0.23	0.55	0	**0.807**	**0.777**	**0.584**
3q1s	113	0	0	0	0.19	0.81	0.15	**0.673**	**0.754**	**0.419**
1p2c	129	**1**	0.05	0	0	0	0	0.68	**0.806**	**0.469**
4ht1	131	0	0	0	0.05	0.14	0.05	**0.622**	**0.59**	**0.21**
3ab0	136	0	0	0	0.33	**0.73**	**0.34**	**0.399**	0.491	-0.068
1v7m	145	0	0	0	0.26	0.77	0.29	**0.902**	**0.792**	**0.685**
4g3y	148	**1**	0.17	0.33	0.03	0.08	0.04	0.705	**0.778**	**0.478**
2vxt	156	0.47	0.36	0.3	0.04	0.09	0.04	**0.648**	**0.692**	**0.337**
3u9p	169	0.06	0.05	0	0.31	**1**	**0.47**	**0.688**	0.695	0.382
3o2d	178	0	0	0	0.32	0.64	0.28	**0.85**	**0.857**	**0.707**
1fns	196	**1**	0.07	0	0	0	0	0.839	**0.727**	**0.555**
3ma9	197	0	0	0	0	0	0	**0.47**	**0.462**	-0.068
3rvv	223	0.15	0.17	0.07	0.25	**0.93**	**0.39**	**0.548**	0.544	0.093
3raj	230	0	0	0	0	0	0	**0.519**	**0.521**	**0.04**
1nfd	239	**0.92**	**0.7**	**0.75**	0.07	0.23	0.04	0.565	0.639	0.19
3i50	273	0	0	0	0	0	0	**0.893**	**0.819**	**0.708**
3gjf	276	0.05	0.11	0.05	0.15	0.66	0.2	**0.64**	**0.759**	**0.381**
3liz	329	0	0	0	0.26	**0.68**	**0.34**	**0.555**	0.51	0.047
3pgf	358	0	0	0	0.02	0.04	0	**0.507**	**0.508**	**0.015**
3zkm	375	0	0	0	0.32	**0.88**	**0.46**	**0.802**	0.684	**0.472**
3r1g	381	0	0	0	0.37	**1**	**0.57**	**0.714**	0.546	0.198
4jr9	409	0.46	0.5	0.46	0.19	0.85	0.34	**0.657**	**0.901**	**0.502**
4ene	442	0	0	0	0	0	0	**0.649**	**0.554**	**0.18**
3o0r	449	0	0	0	0.08	**0.7**	**0.19**	**0.653**	**0.775**	**0.41**
3t3p	453	0.25	0.04	0	0	0	0	**0.599**	**0.696**	**0.278**
1n8z	581	0	0	0	0	0	0	**0.525**	**0.628**	**0.113**

Table S1 The summarizing results on testing set.
